# Neurodiversity and cognitive load in online learning: A focus group study

**DOI:** 10.1371/journal.pone.0301932

**Published:** 2024-04-16

**Authors:** Anne-Laure Le Cunff, Vincent Giampietro, Eleanor Dommett

**Affiliations:** 1 Department of Psychology, Institute of Psychiatry, Psychology & Neuroscience, King’s College London, London, United Kingdom; 2 Department of Neuroimaging, Institute of Psychiatry, Psychology & Neuroscience, King’s College London, London, United Kingdom; Fondazione Policlinico Universitario Gemelli IRCCS, ITALY

## Abstract

It is widely accepted that cognitive load plays a crucial role in online learning. However, despite neurodevelopmental conditions being the largest category of qualifying disabilities in education, and the rise of online learning, there is little understanding of the factors impacting cognitive load in online learning for neurodivergent students and how these factors differ from those affecting neurotypical students. This study used qualitative comparison groups with neurotypical and neurodivergent students to examine their experiences of cognitive load in online learning. A sample of 26 university students (14 neurotypical and 12 neurodivergent) participated in focus group discussions. While neurodivergent students reported many similar experiences of cognitive load in online learning compared to their neurotypical peers—such as confusion in navigating the content and technical issues—some difficulties were more present for neurodivergent students—such as transcripts including mistakes and inaccessible content presentation—creating additional barriers in effectively engaging with the educational content. The results suggest that neurotypical and neurodivergent students experience similar challenges, albeit to differing degrees of intensity, and that more research is needed to explore the relationship between neurodiversity and cognitive load in online learning.

## Introduction

Online learning has become ubiquitous in higher education, with most universities delivering at least part of their curriculum via an online learning platform [1–3]. In 2019, about 35% of all undergraduate students in the United States were enrolled in at least one online course [4]. More recently, the global pandemic has greatly accelerated the transition to online education, altering how instruction is delivered and impacting the learning experience of all students [5]. Effective instructional design is of great importance in the context of online learning, where students have more agency over how they learn [6] and where cognitive barriers, such as lack of attentional control and excessive mind wandering, can have an increased impact on self-regulated learning [7]. Variations in the way individuals experience online education are particularly relevant in the case of students with learning differences associated with neurodiversity, an umbrella term coined in the late 1990s that has come to encompass neurodevelopmental conditions such as autistic spectrum disorder (ASD), attention deficit hyperactivity disorder (ADHD), dyslexia, dyscalculia, dyspraxia, and Tourette syndrome [8, 9]. Neurodivergent people—whose brain functions differ from what is considered typical cognitive and neurodevelopmental functioning—are estimated to represent around 15–20% of the worldwide population [10, 11]. However, as learning differences associated with neurodiversity affect various cognitive processes that are not all easily observed, online learning makes neurodiversity harder to support: without any physical indicators, difficulties are often “hidden”, hampering the implementation of inclusive teaching strategies [12].

One factor that has been extensively studied in the context of online learning more generally is cognitive load, which has been found to impact motivation, focus, and learning [13]. Cognitive load refers to the amount of working memory resources used for the maintenance and processing of information during the completion of a cognitive task [14–16]. Three types of cognitive load have been suggested based on the source of mental effort causing an increase in that specific type of cognitive load: intrinsic load, extraneous load, and germane load [17]. According to the triarchic theory of cognitive load, intrinsic load is linked to the inherent difficulty of a specific topic, extraneous load changes depending on how instruction is delivered to the learner, and germane load varies based on the effort invested by the learner to create a permanent store of knowledge [18]. However, despite its potential importance to improve learning, little is known about how differently neurotypical and neurodivergent students experience cognitive load in online education [19].

Both clinical research and neurodiversity research seek to contribute scientific evidence to lessen impairment experienced by neurodivergent people. While the former focuses on treatment, the latter focuses on adapting environments such as schools and universities to the diverse needs of individuals and ultimately for the benefit of all [20]. In this study, we adopted a participatory research approach to explore cognitive load in online education for both neurotypical and neurodivergent students to understand how online learning environments should be adapted to support all students in higher education. Specifically, we wanted to investigate how neurotypical and neurodivergent students experience cognitive load in online learning, with the view to providing evidence-based guidance for education practitioners. In contrast to the cognitive disability model, we approached neurocognitive differences from a social model perspective, focusing on removing barriers in the online learning environment to enable full engagement by neurodivergent students [21]. The research question was as follows: how do neurotypical and neurodivergent students experience cognitive load in online learning?

## Materials and methods

A qualitative analysis of focus groups was used to draw from participants’ complex personal experiences, perceptions, beliefs, and attitudes through a moderated, semi-structured interaction [22, 23]. Focus groups were selected as a primary data collection method because they capture collective narratives through group dynamics, resulting in richer findings than typically obtained from individual interviews [24]. Considering homogeneous groups tend to yield more focused results, which help understand a particular group in depth, neurotypical and neurodivergent students were assigned to separate groups [23].

Since inclusive research practice has been described as a “requirement of excellence” in neurodiversity research, the study was co-designed in partnership with a Community Advisory Board [25]. Such participatory research involving community partners is conducive to greater external validity and translatability [26, 27]. The protocol, discussion guide, information sheet, and consent form were shared with the Community Advisory Board for feedback and were iteratively improved until ready for submission to the university’s Research Ethics Office. Participants gave written informed consent prior to participation and received a £10 GBP shopping voucher as compensation. All participants expressed their consent for publication. The study was conducted in line with the ethical principles of the Declaration of Helsinki and of the American Psychological Association. The study was approved by the institution’s ethics committee of King’s College London.

Within the context of the present study, the members of the research team considered how their professional background, prior experiences, and assumptions might influence their interactions with participants. Two authors (authors 2 and 3) teach at the institution where the study was hosted, and we were mindful of the potential impact on participants’ willingness to speak candidly about their experiences or how this knowledge might have influenced what was discussed. To mitigate this, author 1, a doctoral candidate and thus a fellow student, facilitated the focus group discussions while remaining cognizant of the power dynamics between the facilitator and the participants, aiming to create a safe and inclusive space where participants’ voices were valued and respected. Furthermore, researchers remained mindful of their biases during data analysis and used iterative peer debriefing as a strategy to minimize the impact of personal factors that could influence the interpretation of the data. However, we recognize that our own assumptions and experiences could still shape how we presented our research findings. To mitigate this, we ensured that our reporting was transparent and strived to represent the participants’ experiences and perspectives accurately by adhering to the Standards for reporting qualitative research (SRQR) checklist [28].

### Participants

Sampling was purposive and included several channels. The study was advertised through the KCL Research Circular, the KCL Neurodiversity & Mental Health Society, and the Researchers And Students on Neurodiversity (ReASoN) Group. All advertisements contained a link to the study information, such that those interested in participating could access the information sheet and consent form before being able to complete a short survey to provide details of demographics, neurodiversity, and study level. This allowed us to ensure that participants met the inclusion criteria and to characterize our overall sample. To be included in the study, all participants had to be English-speaking students over 18 years old enrolled in a university program anywhere in the UK with a present or past online learning experience in a higher education context. For neurodivergent groups, students had to be diagnosed with at least one of the conditions included under the neurodiversity umbrella, namely ADHD, ASD, dyslexia, dyspraxia, dyscalculia, and Tourette syndrome [8]. For neurotypical groups, students needed to be free from any neurodevelopmental condition. To ensure that differences in learning and cognition were due to neurodiversity, both neurodivergent and neurotypical participants had to have no diagnosis of any mental health condition. The sample size was determined by data saturation, which refers to the point at which two consecutive focus groups reveal no additional themes and provide a sufficient understanding of the issues [29]. We aimed for small groups of four to six participants, which are considered appropriate when the goal is to gain in-depth insights into people’s experiences, as they feel safer for participants while retaining fundamental characteristics of focus groups by providing interactive discussions [30].

### Procedure

Focus groups were conducted during the months of May and July 2022. Two modes of participation were offered to provide opportunities for full and equitable participation regardless of personal circumstances or health status: in-person on campus or online via a video conference room. All participants chose to participate in the online version of the focus group discussions. After confirming their preferred time slot, participants received a link to join a one-hour virtual focus group hosted using Microsoft Teams [31]. Based on the feedback we received from the Community Advisory Board and the guidelines for conducting inclusive and accessible focus groups [32, 33], the ground rules and questions were shared both verbally and as a written document, and the participants were allowed to take breaks and to use assistive software. The facilitator used a discussion guide to obtain participants’ perspectives regarding cognitive load in online education (see S1 File). Participants were encouraged to keep their cameras on to simulate in-person interactions as much as possible. While first names were used during the sessions, the transcripts and coded data did not include any names or identifiable information. Data management and storage were handled under the terms of UK data protection law, including the UK General Data Protection Regulation and the Data Protection Act 2018.

### Data analysis

The qualitative data were analyzed using the framework method, which sits within the broad family of methods usually termed thematic analysis. This results in the production of a matrix providing a structure onto which researchers can systematically reduce the data [34]. Framework analysis is often used to methodically describe a population of interest, including the notable variations contained within that population [35]. Framework analysis is considered particularly pertinent in interdisciplinary research because it is more closely aligned to the quantitative paradigm—compared to reflexive methods of thematic analysis—and produces highly structured outputs of summarized data [36, 37]. It consists of five steps: transcription, familiarization, coding, framework development, indexing, charting the data into the framework matrix, and interpreting it [38].

The focus group recordings were transcribed using the computer-assisted qualitative data analysis software (CAQDAS) NVivo [39] to transcribe the discussion with automatically generated timestamps. The email address of each participant was replaced with an ID during the transcription to anonymize the demographic data and the content of the focus groups. Following the “intelligent verbatim” approach to basic transcription, non-lexical sounds (‘ah’, ‘uh’) and repeat words were left out of the transcription [40, 41]. This approach is considered more ethical, as the publication of repetitive or incoherent interview transcripts may lead to the stigmatization of specific groups of people [42].

In the familiarization phase, the resulting transcripts were read several times while capturing reflective notes and sample quotes to explore key themes in the dataset. In the coding phase, patterns and themes in participants’ experiences of cognitive load in online learning were first identified through a combination of inductive coding, consisting in segmenting data into meaningful units of expression and abstracting them into short sequences of words, and deductive coding, using predefined codes based on existing theories [43]. In addition to the sources of cognitive load identified through inductive coding, the domains of cognitive load were coded according to themes from the World Health Organization’s (WHO) International Classification of Functioning, Disability and Health (ICF), specifically from the categories under “Learning and applying knowledge,” such as writing, reading, listening, watching, focusing attention, and making decisions [44].

In the framework development phase, codes were grouped together into the following categories: sources, domains, and moderators of cognitive load in online learning, strategies to manage cognitive load in online learning, and potential improvements to instructional design in online learning. In the indexing phase, the working analytical framework was applied by indexing all transcripts using the existing codes identified in the previous phases. In the charting phase, the data were tabulated based on codes within categories (sources, domains, moderators, strategies, potential improvements) and cases within the participants (neurotypical or neurodivergent), with references to illustrative quotations. Finally, the data were interpreted using a qualitative comparison approach to systematically compare and contrast similarities and differences between the groups [45].

## Results

Saturation of data was reached after conducting six focus groups. Each group had four to six participants, with a total of 26 participants. Our final sample comprised 14 neurotypical students and 12 neurodivergent students, including 15 men and 11 women aged 18–31 (see Table 1). The groups of neurodivergent students included two participants with ASD, four participants with ADHD, three participants with dyslexia, and three participants with ADHD and dyslexia.

**Table 1:** Overview of participants.

Neurotype	Participants	Age	Gender	Study Level	Conditions
Neurotypical	14	Range: 18–28 Median: 23.5	3 women 11 men	Bachelor’s level: 10 Master’s level: 3 Doctoral level: 1	N/A*
Neurodivergent	12	Range: 20–31 Median: 23.5	8 women 4 men	Bachelor’s level: 7 Master’s level: 4 Doctoral level: 1	ASD: 2 ADHD: 4 Dyslexia: 3 ADHD + Dyslexia: 3

*N/A for neurotypical participants indicates no self-reported diagnosis of ADHD, ASD, dyslexia, dyspraxia, dyscalculia, or Tourette’s syndrome specifically, but does not preclude the complete absence of learning differences or other conditions.

**Fig 1:**
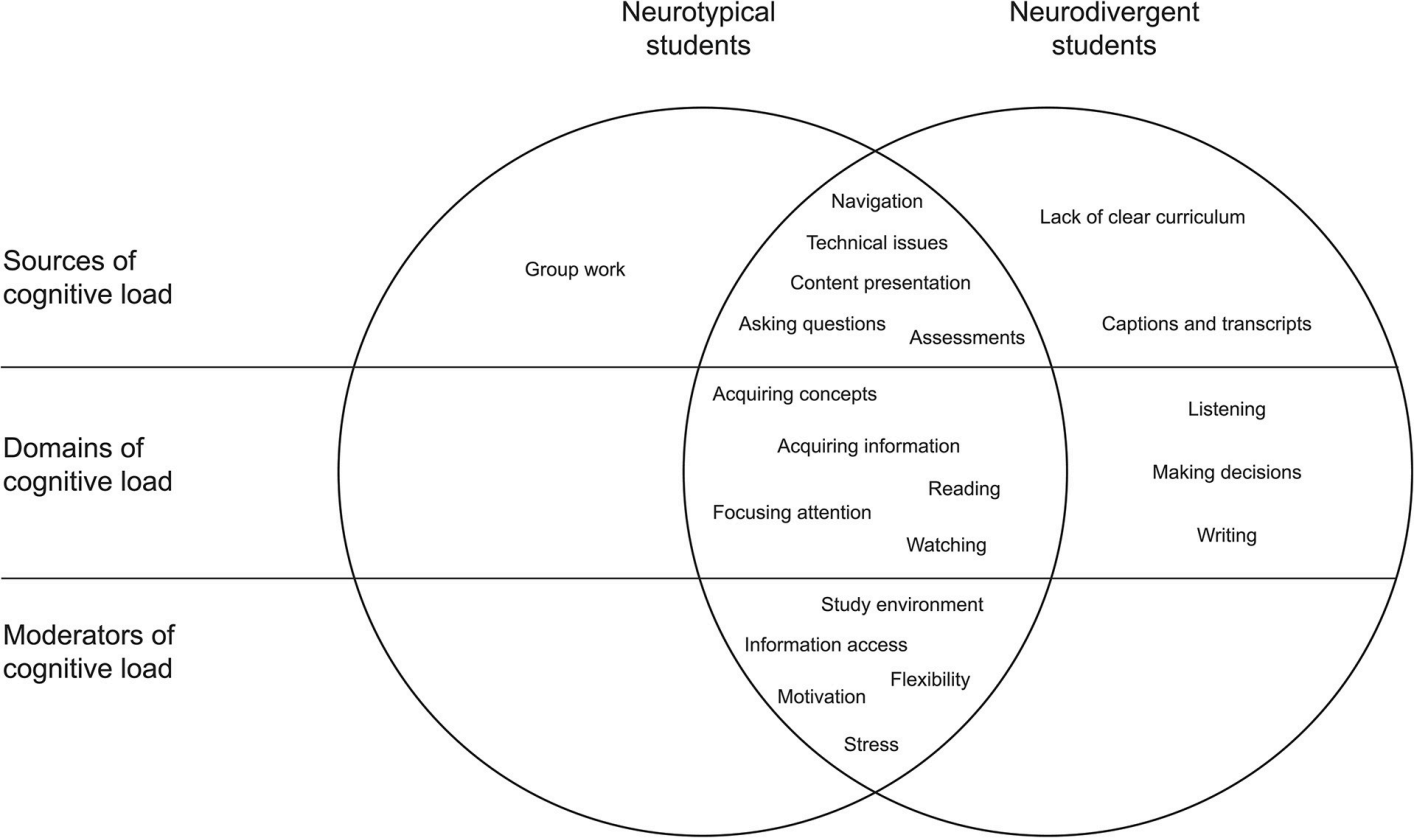
Sources, domains, and moderators of cognitive load in online learning. Venn diagram showing the sources of cognitive load discussed by participants, the domains of cognitive load according to categories from the World Health Organization’s International Classification of Functioning, Disability and Health [43], and the moderators affecting the strength and direction of cognitive load in online learning.

We found both similarities and differences in the way neurodivergent students experienced and managed cognitive load in online learning compared with their neurotypical peers. We categorized these similarities and differences into the following domains: sources of cognitive load, domains of cognitive load, moderators of cognitive load, strategies applied to optimize cognitive load, and suggestions to improve instructional design principles in online learning. The sources, domains, and moderators are shown in Fig 1.

### Sources of cognitive load in online learning

Eight major sources of cognitive load emerged: asking questions, assessments, navigation, technical issues, content presentation, group work, captions and transcripts, and an unclear curriculum. Not all sources of cognitive load were experienced similarly across neurotypes, and some specific aspects of online learning were only challenging for neurotypical or neurodivergent students. We will go through these sources one by one, giving a selection of illustrative quotes.

#### Asking questions

Both neurotypical and neurodivergent students reported experiencing difficulties asking questions during online lectures. However, they gave different reasons for the difficulties. While neurotypical students were frustrated about being unable to ask questions at all when lectures were pre-recorded, some neurodivergent students described difficulty juggling asking questions with taking notes during live lectures.

“I don’t really get to ask questions during recorded sessions.” (P22, Neurotypical)

“Online felt more difficult for me than physically being in university where you can talk to the lecturers if you have any questions, but online, you have to cut someone off. As someone with severe dyslexia, it’s hard for me to pause what I’m wanting to say, write it down, and then say it to someone, because by the time I sorted everything out, I forgot what I was going to say.” (P13, Neurodivergent)

#### Assessments

Assessments were also a source of cognitive load for both neurotypical and neurodivergent students, especially when exams are close together in time, which can make it difficult to plan revision and can cause additional stress.

“I experienced difficulty with the exam prep.” (P19, Neurotypical)

“I think the essays at the end of the year require the most mental effort, and also the deadlines because some of them have the same deadlines in the same day, so that can be overwhelming” (P06, Neurodivergent)

#### Navigation

Neurotypical and neurodivergent students both expressed difficulties in navigating the online learning platform, especially when there were too many tabs, unclear headings, and no tutorial to teach them how to find their way around the system.

“In some of my modules, it was a bit difficult when there were lots of tabs and, and some all had the same headings, and I was trying to find out which one is the one I am specifically looking for. For example, some of the lectures were not titled what the lecture actually was.” (P01, Neurotypical)

“For me, it’s finding my way around all the systems. I find Blackboard not very intuitive and was never really given a tutorial on how to use it. (…) That can be frustrating and can take quite a lot of effort for me to find what I’m looking for and lots of going into the wrong thing and having to click out and try and find stuff again.” (P05, Neurodivergent)

#### Technical problems

Occasional technical problems, such as broken links and connectivity issues, were also mentioned by both neurotypical and neurodivergent students as a source of cognitive load.

“Sometimes when you have technical hiccups, it’s just very annoying. Sometimes they make me drop my working session. When it’s been like 15, 20 minutes. I’m trying to solve the issue and I just get fed up and I lose patience and then I just do something else. And then it requires lots of mental energy to come back to it at a later stage.” (P04, Neurotypical)

“Internet issues, especially lecture seminars being streamed live, and the Internet keeps dropping out and you keep missing things, people can’t hear you or things like that. It just makes it all the more difficult.” (P05, Neurodivergent)

#### Content presentation

While the way content is presented in online learning affects the experience of students across neurotypes, neurodivergent students reported more difficulties in dealing with lectures that were presented too fast, too densely, without any visual aids or enough breaks. Content presentation was reported to affect their ability to focus and to cause confusion, making it difficult to understand and assimilate the content.

“When you go online, the ineffective presentation of the information also causes great problems for me. The way the information or the task is presented is one of my major challenges.” (P17, Neurotypical)

“The way lecturers are delivering the content. Sometimes they give a lot of examples, or they just say too much, more than is really necessary. They make it too dense. That was part of why I found it so enduring.” (P07, Neurodivergent)

“Sometimes they’re just talking over the slides, it’s very boring and it’s hard to stay focused.” (P26, Neurodivergent)

#### Group work

An area that notably differed in terms of cognitive load between neurotypical and neurodivergent students was group work, which seemed to impact neurotypical students more than neurodivergent students. While one neurodivergent student mentioned that it could be harder to coordinate group work in online learning, several neurotypical students described their dissatisfaction with group projects that must be conducted online.

“It was quite hard to try and get everyone together and try and organize something that we can work on and setting up the One Drive folder and the MS Team calls to try and understand all the different ideas and collaborating that way.” (P01, Neurotypical)

“I remember we had a group project, and I was a lot more stressed because you don’t get to see a lot of people in person. How am I going to arrange things to get this done? How are we going to meet? And then, communicating with people. Just finding out who was actually in the group, that was an ordeal as well.” (P02, Neurotypical)

#### Captions and transcripts

Only neurodivergent students raised concerns about the quality of the captions and transcripts in online learning, expressing difficulties in listening to the lecture while keeping up with the automated captions, or finding mistakes in the transcripts, which caused them to waste time trying to find the correct information.

“I can’t keep up with the transcript while listening at the same time. (…) It just takes more effort because you’ve already sat for two hours or whatnot in a lecture. And then you missed something because they either talked too fast or the transcripts are wrong, and you have to go back and be a waste of another two hours where you could just continue.” (P13, Neurodivergent)

#### Curriculum

Similarly, no neurotypical students mentioned the curriculum as a source of cognitive load. In contrast, several neurodivergent students mentioned a lack of clarity in terms of which parts of the curriculum are mandatory for the exams and where to start exactly when being sent a long list of reading materials.

“You have a course and then they upload a long list of books to read without telling you which one to start with. It’s just so full of content, and I think it takes so much time figuring out where to start (…) The teacher uploads so much content and videos and slides, and I don’t know what is mandatory for the exam and what is optional.” (P15, Neurodivergent)

### Domains of cognitive load in online learning

Eight domains of cognitive load were identified based on the themes from the World Health Organization’s (WHO) International Classification of Functioning, Disability and Health (ICF) from the categories under “Learning and applying knowledge”: acquiring concepts, acquiring information, focusing attention, listening, making decisions, reading, watching, and writing [43]. Except for listening, making decisions, and writing, which were only cited by neurodivergent students as domains affected by cognitive load in online learning, all other domains were mentioned across neurotypes. On the other hand, only acquiring concepts and focusing attention were discussed as significant challenges for neurotypical students. Listening, making decisions, reading, watching, focusing, and writing were domains of cognitive load where neurodivergent students reported significant difficulties such as dealing with background noise and overlapping conversations in online lectures, making decisions as to what to study, reading text on a computer screen, sitting through long video lectures, and taking notes (see Table 2 for representative quotes).

**Table 2:** Notable domains of cognitive load for neurodivergent students.

Domains of Cognitive Load	Quotes from Neurodivergent Students
Listening	“When someone’s speaking and then the background noise is there. You can’t even listen to what they’re saying. Or too many people speak at the same time, or when someone asks a really good question and then you’re trying to write it at the same time, and then someone asks another question and you’re just trying to catch up with whatever the other student said.” (P13)
Making Decisions	“It’s demanding in terms of mental effort, just sorting out what is important, and what’s not (…) I’d say for me the most difficult part is deciding what exactly to study.” (P15)
Reading	“I’m severely dyslexic, so reading takes forever even with software because I just don’t have the ability to process it quickly enough.” (P13) “I find it really difficult to read lots of text on a computer screen. So that type of online learning can be really difficult when slides are really text heavy, or I have to read papers that are only available online.” (P05)
Watching/Focusing	“I would say just trying to get through lectures (. . .) They usually are around one hour long, and we had a lot to go through, especially when exams are coming, because of the way they time-tabled it. It was difficult to just get myself to sit down and just go through them.” (P07)
Writing	“Making notes always feels like a lot of effort for me.” (P07)

#### Acquiring concepts

Acquiring concepts was a common issue in neurotypical students, who mentioned the cognitive load involved in “that first step of understanding” and “getting to grips” with new concepts and how it “required a lot of time to get started with something new.” In contrast, only one neurodivergent student mentioned acquiring concepts as a domain of cognitive load in online learning, which did not seem as major a source of distress as other aspects of online learning.

“The part where it’s like a complex concept or something that is quite tricky to understand. To get my head around it or to really get a grasp of it, I have to visualize it in my head or I have to try and understand why this thing happens or where it takes place.” (P01, Neurotypical)

“The last time I studied online was for a project, and there was a lot of new information that I hadn’t seen before. So, it was just getting to grips with that information.” (P19, Neurotypical)

“In some of the lectures, these are new things that come up and it’s a new thing to you, and you have no idea what they’re talking about.” (P14, Neurodivergent)

#### Focusing attention

Both neurotypical and neurodivergent students experienced issues associated with focusing their attention when learning online. However, neurotypical students attributed these challenges to transient situational barriers such as external distractions or low mood. On the other hand, neurodivergent students talked about difficulties in focusing their attention as inherent traits potentially tied to their neurocognitive differences.

“With online study, the hardest part and the one that requires the most effort is staying focused.” (P18, Neurotypical)

“Studying online, alone in your room, you’re not in a good mood sometimes, and sometimes you find it hard to pay attention and focus on what’s going on.” (P10, Neurotypical)

“Some people have neurodiverse conditions where they cannot focus.” (P13, Neurodivergent)

“I’m not sure about other people, but I lose attention very fast.” (P23, Neurodivergent)

#### Acquiring information

Acquiring information was also a shared domain of cognitive load across neurotypes: both neurotypical and neurodivergent students reported difficulties in finding the right information.

“When you do research, you get a whole lot of suggestions and responses from different pages. You have to work your way through what you want to do, and that requires extra effort and extra time.” (P20, Neurotypical)

“I have a challenge when it comes to finding the right information I need (…) Finding the right source and knowing exactly where to start from, I think that’s really been a big deal for me.” (P08, Neurodivergent)

### Moderators of cognitive load in online learning

Several factors emerged as moderators of cognitive load in online learning, namely the study environment, the level of access to information, and the flexibility, motivation, and stress experienced by the student. These moderators affect the strength and direction of the cognitive load in online learning, such as lowering or increasing the students’ perceived cognitive load.

#### Study environment

Participants reported that the study environment had an important impact on their cognitive load in online learning. For neurodivergent students in particular, having control over their environment seemed to enable better cognitive load management by increasing comfort and minimizing distractions.

“There are some environmental constraints sometimes, whether you are on the train or if it’s just you at home.” (P02, Neurotypical)

“It depends on the environment, where you are, like if it’s noisy around you and it’s hard to focus and so on.” (P03, Neurotypical)

“When I’m at home, I have control of the noise and the temperature and I’m alone, so I’m not being distracted by other people and things that might take my focus away from the lecture.” (P05, Neurodivergent)

#### Flexibility

Flexibility appeared to be an important moderator of cognitive load in online learning for both neurotypical and neurodivergent students. The students mentioned the benefits of setting their own schedule and studying at their own pace, wherever and whenever was most convenient.

“You’re able to work out a schedule that’s the most convenient for you.” (P10, Neurotypical)

“That’s one thing I love about online learning, that you can do it at your own pace.” (P14, Neurodivergent)

“I learn at my own pace and in my own time. It’s my time, my mental health, I can study when I’m ready.” (P23, Neurodivergent)

#### Information access

Students across neurotypes discussed information access as another moderator, with easy access to information helping to reduce cognitive load thanks to not worrying about having to take perfect notes during the lectures and being able to go back to the content, or easily access additional information through search engines, online libraries, and other resources on their computer:

“Before online learning, you had to show up at the class and you had to take very careful notes and you were worried that you might have missed something because you didn’t take proper notes. But, online, everything is there.” (P03, Neurotypical)

“Another easy aspect is the fact that everything is just easy to reach. Whatever you need is just there. Whether it’s an online library, or you just search Google, maybe any academic journal you subscribe to, you could just easily have it and access the information you need.” (P25, Neurodivergent)

#### Stress and motivation

In addition to these external factors, two main internal factors seem to moderate cognitive load in online learning for both neurotypical and neurodivergent students: stress and motivation. While stress in online learning seemed to be mostly associated with exams for neurotypical students, neurodivergent students on the other hand described online learning as stressful in and of itself.

“Online is very stressful and at times I can feel bombarded.” (P14, Neurodivergent)

“I often struggle, I waste my mental health and energy and I lose patience.” (P25, Neurodivergent)

Both neurotypical and neurodivergent students described how learning at home on their own requires more self-discipline and self-motivation. It was more difficult to study without the physical presence and support of their teachers and fellow students, especially when the content was not engaging enough.

“It’s hard to motivate yourself at home, you’re on your own, you need to use your own motivation because you don’t have access to the other people, the students, the teachers, and whatnot. They give you motivation when you’re in the same room, but it’s not the same when you study online.” (P10, Neurotypical)

“I feel like I’m not motivated. It’s not even a platform for me to be on because it’s just difficult in general. That’s really it. It’s just mentally overwhelming, it’s exhausting, it just takes so much effort to learn online than it is on campus.” (P13, Neurodivergent)

### Strategies applied to optimize cognitive load in online learning

The main strategies used by students to optimize their cognitive load in online learning were to pause or replay the recording, to plan and manage their time, to seek additional resources, to take breaks, and to take notes. As a reminder, participants in this study were university students reflecting on past or present experiences of online learning in the context of their higher education. As such, they shared strategies across a range of delivery methods, both synchronous virtual meetings and asynchronous recordings.

### Pausing and replaying

Pausing or replaying the content was used by both neurotypical and neurodivergent students to go back to parts of the lesson they did not understand or to take time to digest the information presented in the lesson.

“Being online does give you the option to pause and go back to stuff.” (P02, Neurotypical)

“Being able to pause and take the time to digest what’s on the slides and make any notes and add any reflections makes it a lot less stressful for me.” (P05, Neurodivergent)

#### Planning and managing time

Students across neurotypes reported planning and managing their time by scheduling study time in their calendars, creating lists of tasks, or setting reminders. Those strategies allowed them to study at the most convenient time, consider their other obligations, and not miss any important deadlines.

“I plan an hour if it’s an hour or two hours if it’s two hours, put it in my calendar, just focus and go through the content in one go.” (P03, Neurotypical)

“I create daily tasks and set reminders for myself in order to complete the tasks in a specific window of time.” (P09, Neurotypical)

“When I had to revise, I made a schedule, or I listed the lectures I wanted to get through in a day. That way I know how much I’m going to do, so it doesn’t feel like endlessly going through something.” (P07, Neurodivergent)

“If I prefer to learn in the afternoon or even in the evening or at night, it’s up to me to learn when I’m the most focused and ready to take in all the knowledge.” (P23, Neurodivergent)

“If I have a lot going on, some other projects and even things in my personal life, I can schedule my studies around that.” (P26, Neurodivergent)

#### Seeking additional resources

To optimize their cognitive load, students also seek out additional resources instead of trying to resolve all problems on their own. This strategy includes reaching out to other students, their instructor or supervisor, accessing supplementary material, and looking for other online sources, including YouTube videos.

“When it becomes too difficult, I just try to relate it to someone else, maybe a colleague or a classmate, so we can look into it together and maybe we’ll be able to crack it. And if I still can’t crack it, I just relate it to my supervisor.” (P20, Neurotypical)

“Because some of these things are being recorded, and we can’t really get what we want from it, so we have to source it for ourselves through supplementary material.” (P22, Neurotypical)

“When I don’t get something because the teacher went too quickly through a concept, or they assume things and they don’t really spend enough time to go into the details and I get confused, then I usually go on YouTube.” (P23, Neurodivergent)

#### Taking breaks

While both groups mentioned taking breaks as a way to manage their cognitive load, this strategy seemed crucial for neurodivergent students in online learning. Neurodivergent students reported taking naps, making themselves coffee, or muting the lecture to take a break.

“Online, you can sort of pause it, have a break, and if it’s a hard concept, then you can come back to it when you’re feeling a little bit better, when you’ve kind of refreshed and you can try again to understand.” (P01, Neurotypical)

“You just take a good nap, thirty minutes or one hour, then you come back to the material, and you view it from another perspective.” (P25, Neurodivergent)

“When they have two-hour lectures online, I just take a break. I honestly just can’t concentrate. (…) I’m just trying to take a lot of breaks” (P15, Neurodivergent)

“Online, where I know they’re not going to stop for another two hours, I just mute it and then just go for a break. (…) Some of them don’t even cater for neurodiverse students. So, I have to adapt to how it is. If that means missing some of the lectures, it’s just how it is.” (P13, Neurodivergent)

#### Taking notes

Only neurotypical students mentioned taking notes as a strategy to manage their cognitive load, which is unsurprising given the difficulties taking notes during lectures reported by neurodivergent students.

“Even that motion of just writing it kind of helps me in the sense that it’s not stuck in my head where I’m thinking about it, and I can move on to the next thing.” (P01, Neurotypical)

“When I’m studying online, I try to not just read (…) I try to take notes and then make the meaning easier for me to understand and remember.” (P12, Neurotypical)

### Suggestions to improve the instructional design in online learning

We asked participants what suggestions they had to improve the instructional design in online learning so their cognitive load would be easier to manage. Their suggestions included making the content accessible, offering the content in different formats, giving more breaks, sharing a clear curriculum, systematically recording the lectures, having room for interaction (whether with the instructor or between students), and making sure students can ask questions. Again, as the study sample comprised university students with past or present experiences of online learning as part of their studies, these student-generated recommendations cover diverse online learning modalities within a broader higher education context.

#### Making the content accessible

To make the content more accessible, the students suggested breaking down the content into smaller chunks, having the captions on, ensuring good audio and transcription quality and improving the user experience of the online learning platform.

“With online learning, the most helpful would be to have the captions on, it really helps to try and understand the concepts sometimes. There are some words that I might not know, or sometimes it just helps me to just follow along rather than just looking at the slide by itself.” (P01, Neurotypical)

“Tightening the PowerPoint slides so they match what was said in the lecture. Making sure the captions are accurate and on screen for the lectures helps. If a transcript can be made as well, that would be helpful.” (P01, Neurotypical)

“Splitting recorded videos up into smaller subsections.” (P18, Neurotypical)

“The transcripts need to be changed. I don’t even understand what they’re saying, so afterwards I genuinely want to go back if I really think this is important information. I go back, and the transcripts don’t even match what they’re saying.” (P13, Neurodivergent)

#### Offering a diversity of formats

Another category of suggestions pertaining to offering a diversity of formats to deliver the content, including using various visual formats such as graphics and videos. Neurodivergent students seemed to think that more diverse formats would be most helpful to them.

“As an improvement, you could make it more visual, having photos, pictures, and even sending some videos to students that they can watch when they’re free or in their leisure time.” (P21, Neurotypical)

“Trying to make the activities as different as possible. Maybe something doesn’t have to just be a video of a presentation, that type of thing, because if it’s different, then it can feel more interesting.” (P07, Neurodivergent)

“When writing text, maybe play with colors, play with videos, graphics, and all that.” (P25, Neurodivergent)

“I wish they could do more visualizing. Because, as I said, people do relate more by watching, or by viewing the images, more than reading or the way testing it’s done.” (P24, Neurodivergent)

“Reading some content for hours is quite tiring. I prefer videos a lot more. When the course is well done, it’s more dynamic. You have graphs, you have videos. It all helps break the monotony of just reading a big block of text. I prefer watching videos compared to reading long text.” (P23, Neurodivergent)

#### Giving breaks

Giving more breaks during online lectures would be a welcome improvement for both neurotypical and neurodivergent students, to help them maintain their focus, avoid fatigue, and be more inclusive of all students.

“Sometimes it’s exhausting having a class for a long time. I think having shorter periods of courses for online learning or activities would be better.” (P21, Neurotypical)

“It would also be nice if they could give us breaks from time to time, it shouldn’t always be a stretch lecture.” (P08, Neurodivergent)

#### Sharing a clear curriculum

A clear curriculum with a timetable, deadlines for assignments, and all the resources laid out in advance of the online course or before each online lecture would also help both neurotypical and neurodivergent students better manage their cognitive load in online learning. However, neurodivergent students insisted more on how helpful this particular improvement would be for them.

“It would be quite nice to have a timetable at the beginning of the term with all of those deadlines or lecture release timetables so that I can better plan my time.” (P18, Neurotypical)

“One thing that could be helpful would be to give timings for things like, if there’s a video I need to watch, like how long is it? If there’s a paper that I need to read, how many pages is it? How long is the lecture recording? So that then I can almost prepare myself for the cognitive load that might come along with it.” (P05, Neurodivergent)

“Having clear organization, or maybe like an order for the content, even if it’s just suggested.” (P07, Neurodivergent)

“If I had a magic wand, I would get all the materials in advance. The transcripts, the reading, and make it very clear what’s the best way to prepare for the lecture.” (P16, Neurodivergent)

#### Systematically recording the lectures

Neurodivergent students mentioned that they would ideally like to systematically be able to access the recording of the online lecture after each session, which would allow them to review the content again after the live online lectures.

“I would like them [the teaching staff] to let us record the online lectures. So, you can go into Teams and then press the recorded lecture button.” (P06, Neurodivergent)

“My professor recorded it, so it’s much easier for me to go back and then I was able to get the answers I wanted.” (P13, Neurodivergent)

#### Having room for interaction

Having more room for interaction was an important improvement suggested by students across neurotypes, including interaction with their teacher and interaction among themselves, which would allow for instructors to address the students’ concerns, monitor their progress, and make the online learning experience more engaging.

“One of the solutions that could ease the online learning process might be if after every class there is also a room where the teacher can check back and revisit any question.” (P17, Neurotypical)

“There should be room for interaction. You can also give room for the students to interact within themselves.” (P14, Neurodivergent)

#### Taking and answering questions

Students across neurotypes wanted more opportunities to ask questions and get them answered, whether as part of a group Q&A, by publishing a list of frequently asked questions, or by offering one-to-one sessions for students to ask questions.

“Just making sure that there’s a place to ask questions online or someone to contact if anything arises.” (P01, Neurotypical)

“If a lecture is to last for two hours, there should be an avenue for the students to be able to ask questions.” (P14, Neurodivergent)

“I would say they make a box so we can ask our questions and then they can make it accessible for us to meet them one-on-one online and ask these questions directly.” (P08, Neurodivergent)

## Discussion

While all students across neurotypes were experiencing cognitive load in online learning, the strength and direction of sources, domains, and moderators of cognitive load varied depending on whether the students identified as neurotypical or neurodivergent. To some extent, neurotypical students mentioned more challenges related to intrinsic load—i.e., working memory capacity taken up by the inherent difficulty of the material itself, which is influenced by prior knowledge of the topic. On the other hand, neurodivergent students discussed more difficulties related to extraneous load—i.e., working memory capacity taken up by processes not conducive to learning. For instance, neurotypical students discussed to a larger extent the difficulties they had in understanding the content itself, especially when presented with new, complex information, while neurodivergent students insisted on the impact ineffective captions, transcripts, and content presentation had on their cognitive load. Previous studies suggest that incidental processing due to confusing content presentation and redundant information can lead to cognitive overload [45]. In line with these findings, a potential explanation for the discrepancy between neurotypical and neurodivergent students is that accessibility-related difficulties in online learning (extraneous load) may overtax the available working memory capacity of neurodivergent students. This could result in cognitive overload where neurodivergent students do not have sufficient working memory capacity left to deal with the difficulty of the material itself (intrinsic load).

The domains of cognitive load also highly differed between neurotypical and neurodivergent students. Neurodivergent students reported more difficulties with reading, listening, watching, and writing when engaged in online learning. Reading long-form content without any visual aids was particularly challenging for neurodivergent students, who expressed that it required much effort to stay focused when reading text on a screen, whether it was on a computer or a tablet, and that they often had to re-read the same portion of text many times before they could grasp its content. Such difficulties are unsurprising: reading difficulties are not only core characteristics of dyslexia; they have also been observed across ADHD and ASD [46–49]. Watching long lectures also led to a high level of cognitive load for neurodivergent students, with difficulties in getting themselves to sit down and go through the videos, resulting in tiredness, stress, and lack of focus. Those difficulties with long video lectures may not be linked to the video medium itself but rather to the content presentation, as neurodivergent students also described the benefits of YouTube videos as a complementary format when trying to understand concepts that were not sufficiently explained in the online lectures provided by their university. In fact, several neurodivergent participants suggested using a variety of formats—including videos—to make online learning more engaging. Though there is a dearth of research investigating the impact of video on learning in autistic students, evidence suggests that dyslexic students tend to appreciate the use of visual material and videos in online learning and that a visual presentation of learning contents is effective in enhancing both selective and sustained attention in students with ADHD [50–52]. This suggests that visual and video materials, when used strategically and in an accessible manner, could enhance the learning experience of neurodivergent students.

Taking notes while learning online was difficult for neurodivergent students, a domain of cognitive load not mentioned by neurotypical students. Note-taking difficulties have been found in ADHD [53, 54], ASD [55, 56], and dyslexia [57, 58]. Similar to listening—where background noises, the teacher talking too fast, and other students interrupting the lesson with questions led to extraneous cognitive load—the note-taking difficulties seemed to also arise from competing sensory inputs and modalities of engagement with the content, which made it hard to focus. Atypical sensory processing is a common feature of ASD [59]. Reading, listening, and writing at the same time may have increased the cognitive load of neurodivergent students to a point where taking notes in online learning became overwhelming and stressful.

Despite these dissimilarities, there were many commonalities between neurotypical and neurodivergent students regarding the sources, domains, and moderators of cognitive load in online learning. Focusing their attention was a common challenge across neurotypes, with mentions of internal distractions (not being in the mood for studying, inherent difficulties in maintaining concentration) and external distractions (background noise, being distracted by people around them). While “distracted learning” is not new, it is thought to be exacerbated in online learning [60, 61, 70]. However, neurotypical students predominantly attributed those difficulties to the online learning environment itself, while neurodivergent students tended to discuss these difficulties in relation to individual traits that might hamper their ability to focus. These different perspectives could be explored further in future studies, investigating whether considering attentional difficulties as extrinsic or intrinsic impacts how receptive students are towards changes in their online learning environment.

Participants’ reports of having trouble staying focused for long periods of time in online learning may be linked to motivation, a key moderator of cognitive load [62, 63]. Both neurotypical and neurodivergent students reported that online learning required higher levels of self-discipline to get themselves to study in the absence of the instructor and fellow students in the same room. Without the physical presence of other people, students have to rely on self-motivation alone to study and stay focused when learning on their own.

Also related to focus and motivation, a common moderator of cognitive load for both neurotypical and neurodivergent students is the flexibility offered by online learning, which allows them to study when it is most convenient or when they feel the most motivated. Students across neurotypes described how they appreciated being able to study at their own pace, wherever and whenever was most convenient. Research indeed suggests that a higher level of agency improves students’ learning experience in online learning, though it is unclear whether it positively impacts their learning performance [64, 65]. In addition, not having to commute to go on campus reduced their level of tiredness, also making it easier overall to study. In general, the environment in which the students study online is an important moderator of cognitive load. Having control over their study environment allows students to minimize external stimuli and interruptions, increase focus, and better engage with the content [66, 67]. Neurodivergent students also found it helpful to be able to design a comfortable study environment for themselves, whether it consisted in controlling the noise or the temperature, which in the case of autistic students may be linked to the differences in sensory processing mentioned earlier [59]. On the other hand, a hectic environment can increase extraneous cognitive load, with external distractions such as background noises being particularly disruptive to the online studies of neurodivergent students. Both neurotypical and neurodivergent students reported experiencing stress in online learning, though the stress seemed more targeted around exams for neurotypical students and more diffuse for neurodivergent students. Though research is limited, neurodivergent students are indeed thought to experience significantly higher anxiety around exams [68–70].

Students across neurotypes also had in common several strategies to manage their cognitive load in online learning. Both neurotypical and neurodivergent students frequently paused or replayed the recording, which was reported as an advantage of online learning. While no relevant research has been conducted with neurodivergent students, it has been found that viewing strategies such as pausing and replaying the video can mediate the relationship between extraneous load and germane load in online learning [71]. While neurotypical students mostly insisted on the benefit of being able to go back to some specific concepts, neurodivergent students appreciated the ability to slow down or speed up the videos so that they could match the speed to their own pace. This was described as helpful in having the time to write down important concepts and making the learning experience less stressful. The different motivations behind the “pause and reply” strategy may be explained by how neurotypical students reported experiencing mostly intrinsic cognitive load (pertaining to the concepts themselves), whereas neurodivergent students experienced more extraneous load (pertaining to the way the concepts are presented). Returning to the concepts may allow neurotypical students to grasp them better, while controlling the speed of the videos allows neurodivergent students to reduce the stress caused by trying to match their writing speed to the instructor’s pace.

Time management has been identified as a factor associated with student preparedness for online learning, with many online learning readiness scales including time management items such as meeting deadlines and managing study time to complete assignments [72–74]. Neurotypical and neurodivergent students in our focus groups proactively planned their studies as a strategy to manage their cognitive load, with no major difference between the two groups. Students plan how much time some tasks should take, put this in their calendar, and set up reminders, considering other obligations as well as their energy levels. Though as we have seen, making decisions as to what exactly to study is a challenge mostly faced by neurodivergent students who struggle to navigate confusing curricula, which may impact their ability to schedule their studies to manage their cognitive load. ADHD, in particular, has been connected with a deficit in the perception of time in children and adults [73, 75], which may impede the efficiency of time management strategies for these students. In contrast, it is currently unclear whether ASD is characterized by a fundamental deficit in time perception [76].

Student agency significantly impacts academic performance, benefitting learning, engagement, and well-being through higher levels of motivation, self-efficacy, and metacognition [77, 78]. Both neurotypical and neurodivergent students displayed a high level of agency by seeking additional resources whenever they needed more support or information to achieve their learning goals. These additional resources included asking questions to fellow students in group chats, seeking help from instructors, colleagues, and supervisors, accessing supplementary materials, and performing online searches to find more information about a particular concept. Beyond the intrinsic understanding of the concepts covered in their online courses, neurodivergent students seemed to view online videos, such as on YouTube, as a more enjoyable format to engage with the concepts, a finding that is partly supported by previous research into ADHD and the affinity for multimedia content [50–52].

Taking breaks and taking notes were two strategies where neurotypical and neurodivergent students seemed to differ significantly in terms of application and perception. Although mentioned by some neurotypical students as well, taking breaks was overwhelmingly popular with neurodivergent students to manage their cognitive load. Taking non-screen breaks has been found to help maintain energy levels and engagement in online learning, particularly during longer sessions [79, 80]. Taking breaks is particularly helpful for neurodivergent students who may struggle with maintaining their attention levels for longer periods of time or may feel overwhelmed after some time because of sensory differences [81–83]. Notably, note-taking was only mentioned by neurotypical students, who described it as a way to better understand and remember the content while offloading some of the mental effort of keeping the information in mind so they can move on to the next point. In contrast, neurodivergent students referred to note-taking as an additional source of mental effort, which could be slightly mitigated by typing their notes out, but that was still not considered a helpful strategy to manage their cognitive load. Those difficulties with note-taking could be linked to writing impairments which are common in ADHD and dyslexia and can be found in ASD as well [53–58].

Most of these findings are aligned with existing high-level principles in online learning and education in general, which are often known by instructors but not always applied due to a lack of resources. As neurotypical and neurodivergent students seem to face largely similar challenges when managing their cognitive load in online learning (see Fig 1), our results suggest that the implementation of simple educational strategies to improve focus, reduce stress, and increase interaction in online learning may be effective in improving the experience of students without the need to invest in complex redesigns. Whether these changes would increase learning performance also remains to be investigated, but the investment would be minimal compared to the deployment of more comprehensive frameworks.

Given our aim to provide a rich understanding of cognitive in online learning for both neurotypical and neurodivergent students, our sample size and use of comparison groups were consistent with the qualitative methodology used in studies with a similar design [44]. However, the interpretation of these results is subject to certain limitations.

Comparison groups of neurotypical and neurodivergent students were formed based on prior diagnosis. A limitation of this approach is that neurocognitive profiles likely exist along a spectrum and students without diagnoses may still exhibit traits of neurodivergence [20, 26]. Similarly, co-occurring mental health conditions such as anxiety and depression are common in neurodivergent individuals [84, 85], yet were excluded here to isolate the experiences thought to be most associated with neurodiversity itself. This may limit transferability, as many students have overlapping diagnoses [55, 86]. Future studies could benefit from exploring cognitive load in online learning across a wider range of neurocognitive profiles.

In addition, undergraduate and graduate neurodivergent students were grouped together in the analysis. While the aim of this study was to elucidate shared experiences of cognitive load across the neurodivergent student population, different educational contexts between study levels may shape distinct experiences of cognitive load [87]. Valuable insights could emerge from purposefully sampling undergraduate and graduate neurodivergent students separately in future work. Researchers should thoughtfully consider sampling procedures to match research aims, and whether within-group distinctions are appropriate to introduce based on the guiding inquiry [88].

Another limitation was the gender imbalance in our neurotypical and neurodivergent groups of participants, with most men in the neurotypical group and twice as many women as men in the neurodivergent group. This contradicts the gender ratios observed in research with neurodivergent participants. For instance, men are more likely to be diagnosed with ADHD than women, with a men-to-women ratio of approximately 4:1 in community samples [89]. For ASD, the men-to-women ratio is closer to 3:1 [90]. The gender gap may be less pronounced in dyslexia, with men-to-women ratios ranging from 1.5:1 to 3.3:1 [91]. To address these limitations, care should be taken to recruit a diverse sample of participants who are representative of the larger population, particularly in regard to gender ratios, which are unbalanced in many neurodevelopmental conditions included under the neurodiversity umbrella; future research should be conducted with samples that are more representative of the men-to-women ratio found in the corresponding conditions [89–92]. The findings of the present study could also be strengthened by exploring the relationship between neurodiversity and cognitive load in online learning with a larger sample, for instance, by applying an exploratory-sequential approach to use the qualitative findings from the focus groups to develop a quantitative instrument [93, 94].

## Conclusion

Neurodivergent students reported many similar experiences of cognitive load in online learning compared to their neurotypical peers, such as difficulties in asking questions, stress caused by assessments, confusion in navigating the content, and having to deal with technical issues. However, some challenges were more present for neurodivergent students, such as transcripts including mistakes, inaccessible content presentation, and unclear curricula, causing additional stress and difficulties in effectively engaging with the educational content. The domains of cognitive load in online learning also differed across neurotypes, with neurodivergent students struggling with domains such as listening, writing, reading, listening, watching, and making decisions. In contrast, neurotypical students reported having more trouble understanding concepts. One domain that emerged as a challenge for both neurotypical and neurodivergent students was focusing their attention. Moderators that could positively or negatively impact cognitive load in online learning included the study environment, the flexibility of the online course, information access, and their levels of stress and motivation. Students used various strategies to manage their cognitive load in online learning, such as pausing or replaying the recording, planning their study time, seeking additional resources outside of the core material, and taking breaks. Taking breaks was particularly important for neurodivergent students, who felt overwhelmed by long online lectures. Notably, taking notes was only mentioned by neurotypical students as a way to manage their cognitive load in online learning. Overall, the results suggest that neurotypical and neurodivergent students face similar challenges with regard to cognitive load in online learning, albeit at varying levels of intensity. Inclusive design practices may benefit all students.

## Supporting information

S1 File Focus groups discussion guide. (PDF)

## References

[1] Morris NP, Ivancheva M, Coop T, Mogliacci R, Swinnerton B. Negotiating growth of online education in higher education. International Journal of Educational Technology in Higher Education. 2020 Dec;17:1–6. doi: 10.1186/s41239-020-00227-w

[2] Picciano AG. Online learning: Implications for higher education pedagogy and policy. Journal of Thought. 2006 Apr 1;41(1):75–94.

[3] Singh V, Thurman A. How many ways can we define online learning? A systematic literature review of definitions of online learning (1988–2018). American Journal of Distance Education. 2019 Oct 2;33(4):289–306. doi: 10.1080/08923647.2019.1663082

[4] De Brey C, Snyder TD, Zhang A, Dillow SA. Digest of Education Statistics 2019. NCES 2021–009. National Center for Education Statistics. 2021 Feb.

[5] Kumar K, Pande BP. Rise of online teaching and learning processes during COVID-19 pandemic. Predictive and preventive measures for COVID-19 pandemic. 2021:251–71. doi: 10.1007/978-981-33-4236-1_14

[6] Song L, Hill JR. A conceptual model for understanding self-directed learning in online environments. Journal of Interactive Online Learning. 2007 Mar 1;6(1):27–42.

[7] Kohan N, Arabshahi KS, Mojtahedzadeh R, Abbaszadeh A, Rakhshani T, Emami A. Self-directed learning barriers in a virtual environment: a qualitative study. Journal of advances in medical education & professionalism. 2017 Jul;5(3):116. 28761885 PMC5522903

[8] Clouder L, Karakus M, Cinotti A, Ferreyra MV, Fierros GA, Rojo P. Neurodiversity in higher education: A narrative synthesis. Higher Education. 2020 Oct;80(4):757–78. doi: 10.1007/s10734-020-00513-6

[9] Singer J. Why can’t you be normal for once in your life? From a problem with no name to the emergence of a new category of difference. Disability discourse. 1999:59–70.

[10] Doyle N. Neurodiversity at work: a biopsychosocial model and the impact on working adults. British Medical Bulletin. 2020 Sep;135(1):108. doi: 10.1093/bmb/ldaa021 32996572 PMC7732033

[11] Jurgens A. Neurodiversity in a neurotypical world: An enactive framework for investigating autism and social institutions. In Neurodiversity Studies 2020 Jun 2 (pp. 73–88). Routledge.

[12] Matthews N. Teaching the ‘invisible’ disabled students in the classroom: disclosure, inclusion and the social model of disability. Teaching in higher education. 2009 Jun 1;14(3):229–39. doi: 10.1080/13562510902898809

[13] Skulmowski A, Xu KM. Understanding cognitive load in digital and online learning: A new perspective on extraneous cognitive load. Educational psychology review. 2022 Mar. doi: 10.1007/s10648-021-09624-7

[14] Barrouillet P, Bernardin S, Camos V. Time constraints and resource sharing in adults’ working memory spans. Journal of experimental psychology: General. 2004 Mar;133(1):83. doi: 10.1037/0096-3445.133.1.83 14979753

[15] Barrouillet P, Camos V. The time-based resource-sharing model of working memory. 2020: 85–115. doi: 10.1093/oso/9780198842286.003.0004

[16] Chandler P, Sweller J. Cognitive load theory and the format of instruction. Cognition and instruction. 1991 Dec 1;8(4):293–332. doi: 10.1207/s1532690xci0804_2

[17] Sweller J, Van Merrienboer JJ, Paas FG. Cognitive architecture and instructional design. Educational psychology review. 1998 Sep 1:251–96. doi: 10.1023/A:1022193728205

[18] Sweller J, van Merriënboer JJ, Paas F. Cognitive architecture and instructional design: 20 years later. Educational Psychology Review. 2019 Jun 15;31:261–92. doi: 10.1007/s10648-019-09465-5

[19] Le Cunff AL, Giampietro V, Dommett E. Neurodiversity and cognitive load in online learning: A systematic review with narrative synthesis. Educational Research Review. 2024 Mar 25. doi: 10.1016/j.edurev.2024.100604

[20] Sonuga-Barke E, Thapar A. The neurodiversity concept: is it helpful for clinicians and scientists?. The Lancet Psychiatry. 2021 Jul 1;8(7):559–61. doi: 10.1016/S2215-0366(21)00167-X 33984295

[21] Rosqvist HB, Chown N, Stenning A, editors. Neurodiversity studies: A new critical paradigm. Routledge; 2020 Jun 2.

[22] Kitzinger J. The methodology of focus groups: the importance of interaction between research participants. Sociology of health & illness. 1994 Jan;16(1):103–21. doi: 10.1111/1467-9566.ep11347023

[23] Morgan DL. Focus groups. Annual review of sociology. 1996 Aug;22(1):129–52. doi: 10.1146/annurev.soc.22.1.129

[24] Gill P, Stewart K, Treasure E, Chadwick B. Methods of data collection in qualitative research: interviews and focus groups. British dental journal. 2008 Mar 22;204(6):291–5. doi: 10.1038/bdj.2008.192 18356873

[25] Le Cunff AL, Logan PE, Ford R, Martis BL, Mousset I, Sekibo J, et al. Co-design for participatory neurodiversity research: collaborating with a community advisory board to design a research study. Journal of Participatory Research Methods. 2023 Feb 2;4(1).

[26] Fletcher-Watson S, Brook K, Hallett S, Murray F, Crompton CJ. Inclusive practices for neurodevelopmental research. Current Developmental Disorders Reports. 2021 Jun;8:88–97. doi: 10.1007/s40474-021-00227-z PMC11832739 39974555

[27] Lasker RD, Weiss ES. Broadening participation in community problem solving: a multidisciplinary model to support collaborative practice and research. Journal of Urban Health. 2003 Mar;80:14–47. doi: 10.1093/jurban/jtg014 12612096 PMC3456118

[28] O’Brien BC, Harris IB, Beckman TJ, Reed DA, Cook DA. Standards for reporting qualitative research: a synthesis of recommendations. Academic medicine. 2014 Sep 1;89(9):1245–51. doi: 10.1097/ACM.0000000000000388 24979285

[29] Hennink MM, Kaiser BN, Weber MB. What influences saturation? Estimating sample sizes in focus group research. Qualitative health research. 2019 Aug;29(10):1483–96. doi: 10.1177/1049732318821692 30628545 PMC6635912

[30] Krueger RA. Participants in Focus Groups. Focus groups: A practical guide for applied research. Sage publications; 2014 Aug 14.

[31] Microsoft Corporation. Microsoft Teams. Microsoft; 2021.

[32] Nind M. Conducting qualitative research with people with learning, communication and other disabilities: Methodological challenges. 2008.

[33] Wattenberg TL. Online focus groups used as an accessible participatory research method. In Proceedings of the 7th International ACM SIGACCESS Conference on Computers and Accessibility 2005 Oct 9 (pp. 180–181).

[34] Ritchie J, Lewis J, Nicholls CM, Ormston R, editors. Qualitative research practice: A guide for social science students and researchers. sage; 2013 Nov 1.

[35] Goldsmith LJ. Using Framework Analysis in Applied Qualitative Research. Qualitative Report. 2021 Jun 1;26(6). doi: 10.46743/2160-3715/2021.5011

[36] Gale NK, Heath G, Cameron E, Rashid S, Redwood S. Using the framework method for the analysis of qualitative data in multi-disciplinary health research. BMC medical research methodology. 2013 Dec;13(1):1–8. doi: 10.1186/1471-2288-13-117 24047204 PMC3848812

[37] Pope C, Mays N. Critical reflections on the rise of qualitative research. Bmj. 2009 Sep 15;339. doi: 10.1136/bmj.b3425

[38] QSR International Pty Ltd. NVivo. 2020.

[39] Bucholtz M. The politics of transcription. Journal of pragmatics. 2000 Sep 1;32(10):1439–65. doi: 10.1016/S0378-2166(99)00094-6

[40] McMullin C. Transcription and Qualitative Methods: Implications for Third Sector Research. VOLUNTAS: International Journal of Voluntary and Nonprofit Organizations, 2021:1–14. doi: 10.1007/s11266-021-00400-3 34522070 PMC8432276

[41] Kvale S. Interviews: An introduction to qualitative research interviewing. Sage Publications, Inc; 1994. doi: 10.1163/156916294X00016

[42] Linneberg MS, Korsgaard S. Coding qualitative data: A synthesis guiding the novice. Qualitative research journal. 2019 May 8.

[43] Üstün TB, Chatterji S, Bickenbach J, Kostanjsek N, Schneider M. The International Classification of Functioning, Disability and Health: a new tool for understanding disability and health. Disability and rehabilitation. 2003 Jan 1;25(11–12):565–71. doi: 10.1080/0963828031000137063 12959329

[44] Lindsay S. Five approaches to qualitative comparison groups in health research: a scoping review. Qualitative health research. 2019 Feb;29(3):455–68. doi: 10.1177/1049732318807208 30501574

[45] Elliott SN, Kurz A, Beddow P, Frey J. Cognitive load theory: Instruction-based research with applications for designing tests. In Proceedings of the National Association of School Psychologists’ Annual Convention, Boston, MA, February 2009 Feb 24 (Vol. 24, pp. 1–22).

[46] Fletcher JM. Dyslexia: The evolution of a scientific concept. Journal of the International Neuropsychological Society. 2009 Jul;15(4):501–8. doi: 10.1017/S1355617709090900 19573267 PMC3079378

[47] Henderson LM, Clarke PJ, Snowling MJ. Reading comprehension impairments in autism spectrum disorders. L’Année psychologique. 2014 Dec 1;114(4):779–97. doi: 10.3917/anpsy.144.0779

[48] Paloyelis Y, Rijsdijk F, Wood AC, Asherson P, Kuntsi J. The genetic association between ADHD symptoms and reading difficulties: the role of inattentiveness and IQ. Journal of abnormal child psychology. 2010 Nov;38:1083–95. doi: 10.1007/s10802-010-9429-7 20556504 PMC2964469

[49] Parks KM, Moreau CN, Hannah KE, Brainin L, Joanisse MF. The task matters: A scoping review on reading comprehension abilities in ADHD. Journal of Attention Disorders. 2022 Aug;26(10):1304–24. doi: 10.1177/10870547211068047 34961391

[50] Antonietti A, Fabio RA, Iannello P, Zugno E. Multimedia learning in ADHD students. In Attention-Deficit Hyperactivity Disorder: Diagnosis, prevalence and treatment 2021 (pp. 71–95). Nova Science Publishers.

[51] Barnett JE. Helping students with ADHD in the age of digital distraction. Research, Advocacy, and Practice for Complex and Chronic Conditions. 2017 Dec 30;36(2):1–7. doi: 10.14434/pders.v36i2.23913

[52] Smith CF. Advanced undergraduate students with dyslexia: perceptions of social supports that buffer college-related stress and facilitate academic success. Southern Connecticut State University; 2017.

[53] Gleason JD. An Investigation of the Note-taking Skills of Adolescents with and without Attention Deficit Hyperactivity Disorder (ADHD): An Extension of Previous Research (Doctoral dissertation, Columbia University). 2012 Feb 8. doi: 10.7916/D8WS9170

[54] Vekaria PC, Peverly ST. Lecture note-taking in postsecondary students with attention-deficit/hyperactivity disorder. Reading and Writing. 2018 Sep;31:1551–73.

[55] Cai RY, Richdale AL. Educational experiences and needs of higher education students with autism spectrum disorder. Journal of autism and developmental disorders. 2016 Jan;46:31–41. doi: 10.1007/s10803-015-2535-1 26216381

[56] Reed DK, Hallett A, Rimel H. Note-taking instruction for college students with autism spectrum disorder. Exceptionality. 2016 Oct 1;24(4):195–212.

[57] Fuller M, Healey M, Bradley A, Hall T. Barriers to learning: a systematic study of the experience of disabled students in one university. Studies in higher education. 2004 Jun 1;29(3):303–18.

[58] Mortimore T, Crozier WR. Dyslexia and difficulties with study skills in higher education. Studies in higher education. 2006 Apr 1;31(2):235–51.

[59] Marco EJ, Hinkley LB, Hill SS, Nagarajan SS. Sensory processing in autism: a review of neurophysiologic findings. Pediatric research. 2011 May;69(8):48–54. doi: 10.1203/PDR.0b013e3182130c54 21289533 PMC3086654

[60] Dontre AJ. The influence of technology on academic distraction: A review. Human Behavior and Emerging Technologies. 2021 Jul;3(3):379–90. doi: 10.1002/hbe2.229

[61] Schmidt SJ. Distracted learning: Big problem and golden opportunity. Journal of Food Science Education. 2020 Oct;19(4):278–91. doi: 10.1111/1541-4329.12206

[62] Abeysekera L, Dawson P. Motivation and cognitive load in the flipped classroom: definition, rationale and a call for research. Higher education research & development. 2015 Jan 2;34(1):1–4. doi: 10.1080/07294360.2014.934336

[63] Schnotz Fries, Horz. Motivational aspects of cognitive load theory. Wosnitza, Karabenick, Efklides, Nenniger, editors. Contemporary Motivation Research: From Global to Local Perspectives. 2009 Jan 1;69–96.

[64] Prakasha GS, Sarah HH, Hemalathaa KY. Examining learner agency in online teaching. Universal Journal of Educational Research. 2020;8(12):6509–16. doi: 10.13189/ujer.2020.081216

[65] Xie B, Nelson GL, Akkaraju H, Kwok W, Ko AJ. The effect of informing agency in self-directed online learning environments. In Proceedings of the Seventh ACM Conference on Learning@ Scale 2020 Aug 12 (pp. 77–89). doi: 10.1145/3386527.3405928

[66] Choi HH, Van Merriënboer JJ, Paas F. Effects of the physical environment on cognitive load and learning: Towards a new model of cognitive load. Educational Psychology Review. 2014 Jun;26:225–44. doi: 10.1007/s10648-014-9262-6

[67] Jafari MJ, Khosrowabadi R, Khodakarim S, Mohammadian F. The effect of noise exposure on cognitive performance and brain activity patterns. Open access Macedonian journal of medical sciences. 2019 Sep 9;7(17):2924. doi: 10.3889/oamjms.2019.742 31844459 PMC6901841

[68] Carroll JM, Iles JE. An assessment of anxiety levels in dyslexic students in higher education. British journal of educational psychology. 2006 Sep;76(3):651–62. doi: 10.1348/000709905X66233 16953967

[69] Nelson JM, Lindstrom W, Foels PA. Test anxiety and college students with attention deficit hyperactivity disorder. Journal of Psychoeducational Assessment. 2014 Sep;32(6):548–57. doi: 10.1177/0734282914521978

[70] Zavery A, Zäch M, Bertrams A. Test Anxiety in Autistic University Students—Preliminary Results from a German-Speaking Sample. Brain Sciences. 2021 Mar 19;11(3):390. doi: 10.3390/brainsci11030390 33808816 PMC8003700

[71] Costley J, Fanguy M, Lange C, Baldwin M. The effects of video lecture viewing strategies on cognitive load. Journal of Computing in Higher Education. 2021 Apr;33:19–38. doi: 10.1007/s12528-020-09254-y

[72] Martin F, Stamper B, Flowers C. Examining Student Perception of Readiness for Online Learning: Importance and Confidence. Online Learning. 2020 Jun;24(2):38–58. doi: 10.24059/olj.v24i2.2053

[73] Smith A, Taylor E, Warner Rogers J, Newman S, Rubia K. Evidence for a pure time perception deficit in children with ADHD. Journal of child psychology and psychiatry. 2002 May;43(4):529–42. doi: 10.1111/1469-7610.00043 12030598

[74] Zimmerman WA, Kulikowich JM. Online learning self-efficacy in students with and without online learning experience. American Journal of Distance Education. 2016 Jul 2;30(3):180–91. doi: 10.1080/08923647.2016.1193801

[75] Antshel KM, Hier BO, Barkley RA. Executive functioning theory and ADHD. In Handbook of executive functioning 2013 Sep 12 (pp. 107–120). New York, NY: Springer New York. doi: 10.1007/978-1-4614-8106-5_7

[76] Casassus M, Poliakoff E, Gowen E, Poole D, Jones LA. Time perception and autistic spectrum condition: A systematic review. Autism Research. 2019 Oct;12(10):1440–62. doi: 10.1002/aur.2170 31336032 PMC6852160

[77] Reeve J. How students create motivationally supportive learning environments for themselves: The concept of agentic engagement. Journal of educational psychology. 2013 Aug;105(3):579. doi: 10.1037/a0032690

[78] Xiao J. Learner agency in language learning: The story of a distance learner of EFL in China. Distance Education. 2014 Jan 2;35(1):4–17. doi: 10.1080/01587919.2014.891429

[79] Khan RA, Atta K, Sajjad M, Jawaid M. Twelve tips to enhance student engagement in synchronous online teaching and learning. Medical Teacher. 2022 Jun 3;44(6):601–6. doi: 10.1080/0142159X.2021.1912310 33877950

[80] Pacheco E. Twelve tips for online live classes. MedEdPublish. 2020 Nov 10;9(250):250. doi: 10.15694/mep.2020.000250.1

[81] Brock SE, Puopolo M, Cummings C, Husted D. ADHD: Classroom interventions. Helping children at home and school III: Handouts from your school psychologist. 2010:S8H5.

[82] Goodall C. ‘I felt closed in and like I couldn’t breathe’: A qualitative study exploring the mainstream educational experiences of autistic young people. Autism & Developmental Language Impairments. 2018 Sep;3:2396941518804407. doi: 10.1177/2396941518804407

[83] Jones EK, Hanley M, Riby DM. Distraction, distress and diversity: Exploring the impact of sensory processing differences on learning and school life for pupils with autism spectrum disorders. Research in autism spectrum disorders. 2020 Apr 1;72:101515. doi: 10.1016/j.rasd.2020.101515

[84] Mosner MG, Kinard JL, Shah JS, McWeeny S, Greene RK, Lowery SC, et al. Rates of co-occurring psychiatric disorders in autism spectrum disorder using the mini international neuropsychiatric interview. Journal of autism and developmental disorders. 2019 Sep 15;49:3819–32. doi: 10.1007/s10803-019-04090-1 31175504 PMC6669096

[85] Tannock R. ADHD with anxiety disorders. In: Brown, editor. ADHD comorbidities: Handbook for ADHD complications in children and adults. American Psychiatric Publishing, Inc.; 2009. p. 131–55.

[86] Wolf LE. College students with ADHD and other hidden disabilities: Outcomes and interventions. Annals of the New York Academy of Sciences. 2001 Jun;931(1):385–95. 11462755 10.1111/j.1749-6632.2001.tb05792.x

[87] Hahnel C, Schoor C, Kroehne U, Goldhammer F, Mahlow N, Artelt C. The role of cognitive load in university students’ comprehension of multiple documents. Zeitschrift für pädagogische Psychologie. 2019(2):105–118.

[88] Robinson OC. Sampling in interview-based qualitative research: A theoretical and practical guide. Qualitative research in psychology. 2014 Jan 2;11(1):25–41.

[89] Ramtekkar UP, Reiersen AM, Todorov AA, Todd RD. Sex and age differences in attention-deficit/hyperactivity disorder symptoms and diagnoses: implications for DSM-V and ICD-11. Journal of the American Academy of Child & Adolescent Psychiatry. 2010 Mar 1;49(3):217–28. doi: 10.1097/00004583-201003000-00005 20410711 PMC3101894

[90] Loomes R, Hull L, Mandy WP. What is the male-to-female ratio in autism spectrum disorder? A systematic review and meta-analysis. Journal of the American Academy of Child & Adolescent Psychiatry. 2017 Jun 1;56(6):466–74. doi: 10.1016/j.jaac.2017.03.013 28545751

[91] Rutter M, Caspi A, Fergusson D, Horwood LJ, Goodman R, Maughan B, et al. Sex differences in developmental reading disability: new findings from 4 epidemiological studies. Jama. 2004 Apr 28;291(16):2007–12. doi: 10.1001/jama.291.16.2007 15113820

[92] Shaywitz SE, Shaywitz BA, Fletcher JM, Escobar MD. Prevalence of reading disability in boys and girls: Results of the Connecticut Longitudinal Study. Jama. 1990 Aug 22;264(8):998–1002. doi: 10.1001/jama.1990.03450080084036 2376893

[93] Edmonds WA, Kennedy TD. An applied guide to research designs: Quantitative, qualitative, and mixed methods. Sage Publications; 2016 Apr 20. doi: 10.4135/9781071802779.n18

[94] Tashakkori A, Teddlie C. Sage handbook of mixed methods in social & behavioral research. SAGE publications; 2021 Jun 17.

