# Supplementary material for: Neurodiversity and cognitive load in online learning: A focus group study
Source: PLoS One. 2024 Apr 16;19(4):e0301932. doi: 10.1371/journal.pone.0301932 (PMC11020716; doi:10.1371/journal.pone.0301932)
Supplement: S1 File — (PDF) [file pone.0301932.s001.pdf]

S1 File. Focus Groups Discussion guide.

ID	Question
1	What do you think cognitive load is?
2	Using this definition of cognitive load, what elements of learning do you think require the most load?
3	Think about the last time you had to study online, which aspects did you feel required the most cognitive load?
4	Where you have experienced difficulties, what do you think underpinned them?<br>Potential follow-up prompt: Was it due to the content itself being difficult, the way the content was presented, or the mental effort you were investing into properly understanding the content?
5	Thinking about that same time you were studying online, which aspects did you find easiest?
6	What phase(s) of online learning do you feel require the most cognitive load—learning, collaborating, assessments, or something else?
7	Do you think investing a lot of mental effort is necessary to study online, something helpful, or something to be avoided?
8	Do you apply any strategies to manage your cognitive load when studying online?
9	Is there anything you think instructors could do when designing online courses that would make it easier for you to manage your cognitive load when studying online?
10	Is there anything else you want to share that we haven’t discussed yet?
